# Low hand grip strength as an indicator of depression in the Korean population: a large-scale cross-sectional study

**DOI:** 10.3389/fpubh.2024.1421291

**Published:** 2024-09-12

**Authors:** Bum Ju Lee

**Affiliations:** Digital Health Research Division, Korea Institute of Oriental Medicine, Daejeon, Republic of Korea

**Keywords:** depression, relative hand grip strength, absolute hand grip strength, anthropometry, risk factor

## Abstract

**Background:**

Depression is one of the leading global mental health problems, and hand grip strength (HGS) is associated with depression. However, there have been no studies assessing the association between depression and relative HGS indices combined with waist circumference (WC) and the waist-to-height ratio (WHtR). The objective of this study was to examine the association of depression with absolute and relative HGS indices.

**Methods:**

This was a cross-sectional study based on the Korea National Health and Nutrition Examination Survey from 2014 to 2019. A total of 20,649 participants (8,959 men, 43.4% and 11,690 women, 56.6%) were included. The associations between depression and the HGS indices were analyzed through complex sample binary logistic regression models, which were adjusted for age in Model 1 and various covariates in Model 2.

**Results:**

The prevalence of depression was 4.58%, with rates of 2.29% for men and 6.34% for women. The prevalence of depression in women was 2.76 times greater than that in men. In men, the mean HGS values in the dominant hand were 35.48 ± 0.75 kg in the depression group and 38.73 ± 0.11 kg in the non-depression group; in women, they were 21.37 ± 0.22 kg in the depression group and 22.77 ± 0.07 kg in the non-depression group. In men, relative HGS indices as HGS/WC, HGS/body mass index, and HGS/WHtR were more strongly associated with depression than were the absolute HGS indices; however, in women, the associations were similar for both absolute and relative HGS indices. The magnitude of the association was greater for men than for women. In both sexes, all the anthropometric indices had a lower association with depression than did the HGS indices.

**Conclusion:**

Low absolute and relative HGS were negatively associated with depression in the Korean population. But, relative HGS indices were more strongly associated with depression than were absolute HGS and anthropometric indices in men but not in women.

## 1 Introduction

Depression is a common mental disorder that leads to increased mortality and disability and is related to an increased risk of suicide (1–4). The global prevalence of depression was 4.4% in 2015, and depression was more common in women (5.1%) than in men (3.6%) (2, 3); moreover, the prevalence of clinical symptoms of depression was 6.54% in Europe (4). The prevalence of depressive symptoms is associated with different socioeconomic and sociodemographic characteristics (i.e., income, education level, employment, age, and sex) and different healthy lifestyle features (i.e., healthy diet, alcohol consumption, physical exercise, smoking, obesity, and healthy sleep) (2, 4, 5). These characteristics may induce country or regional variations in the prevalence of depression and may be considered risk factors for depression, except for differences in genetic profiles (2, 4, 5). Biological treatments such as N-methyl-D-aspartate modulators, γ-amino butyric acid modulators, anti-inflammatory agents, and brain stimulation are considered to improve and treat depression (2).

Recently, several studies conducted in several different countries have investigated the association between depression and hand grip strength (HGS) (6–15). These studies have suggested that low HGS is negatively associated with depression (6–13). For example, in a longitudinal study, individuals with lower HGS exhibited greater development of depression symptoms after 1 year (8). Women with middle or high depressive symptoms had a 1.60 kg lower HGS than did those with no or few symptoms (10). Therefore, most studies have shown that HGS indices are useful indicators of depression and depressive symptoms. In addition to studies on the relationship between absolute HGS and chronic diseases, recent studies have assessed the association between chronic diseases and relative HGS, which is calculated by dividing HGS by body mass index (BMI) or weight (16–18). For example, when testing the associations of metabolic syndrome with absolute HGS and relative HGS indices, the relative HGS index was a more powerful predictor of metabolic syndrome than was the absolute HGS index (16). Additionally, both absolute HGS indices and relative HGS indices have similar associations with arthritis and anemia (17, 18). Also, HGS was associated with several clinical characteristics such as cholesterol and immune cell counts, and the associations differed according to age and sex (19). However, there have been no studies examining the association between depression and absolute or relative HGS indices. Therefore, the objective of this study was to examine the association between depression and absolute and relative HGS indices in a Korean population. Because depression is strongly associated with obesity (2), it is important to determine the association between depression and relative HGS calculated by anthropometry.

The originality and significance of the present study are that it is the first to compare the association between depression and absolute and relative HGS indices and to demonstrate that relative HGS indices are more strongly associated with depression than are absolute HGS in men but not in women. To our knowledge, this study is the first to report the usefulness of relative HGS indices combined with WC and WHtR for screening for depression.

## 2 Materials and methods

### 2.1 Study population and data sources

**Figure 1:**
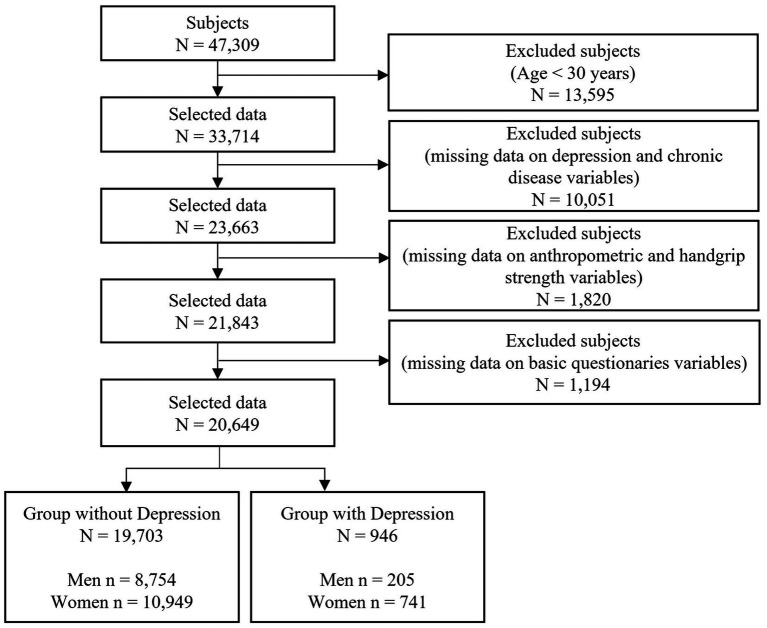
Sample selection process in this study.

The Korea Disease Control and Prevention Agency (KDCA) conducts the Korea National Health and Nutrition Examination Survey (KNHANES) to produce nationally representative and reliable statistics on the health status and conditions of the South Korean population. To achieve this goal, the survey utilizes the sampling framework of the Population and Housing Census and data on joint housing price disclosures. This study utilized health survey and health examination data collected through the KNHANES. Both the health survey and examination were conducted in mobile examination units. The health survey employed interviews and self-administered methods, while the health examination involved direct measurements and laboratory analyses. For this research, data from 2014 to 2019 were utilized, encompassing a total of 47,309 participants (21,566 men and 25,743 women). The participants in this study were adults aged 30 years and older. Through the various inclusion and exclusion procedures depicted in Figure 1, a total of 20,649 participants (8,959 men and 11,690 women) without missing data for the analysis variables were ultimately selected.

The KNHANES was conducted with the approval of the Institutional Review Board (IRB) of the KDCA (IRB: 2013-12EXP-03-5C, 2018-01-03-P-A, 2018-01-03-C-A) (20). Additionally, ethical approval for this study, based on the KNHANES dataset, was obtained from the Institutional Review Board of the Korea Institute of Oriental Medicine (IRB No. I-2209/009–001). Written informed consent was obtained from all participants. This study adhered to the principles of the Helsinki Declaration, and all the analytical methods were performed in accordance with the guidelines of the KDCA (20–22).

### 2.2 Definitions of depression

Depression was defined based on responses to the question posed during a health interview survey: “Do you have depression diagnosed by a doctor?” Subjects who responded “yes” were categorized into the depression group, while those who responded “no” were included in the non-depression group. This question was administered through face-to-face health interviews conducted by trained personnel who adhered to the guidelines for each item rather than relying on self-diagnostic questionnaires (20–22).

### 2.3 Measurement

In this study, we analyzed the association between depression and variables related to HGS and anthropometric measurements. HGS was measured using a digital grip strength dynamometer (T.K.K 5401, Japan; Takei Scientific Instruments Co., Ltd., Tokyo, Japan). During HGS measurement, subjects who were experiencing functional limitations or discomfort, such as arm, hand, or finger deformities; fractures; recent hand/wrist surgery within the past 3 months; or recent pain in the hand within the last 7 days, were excluded. The measurement was conducted with the subject in a standing position, maintaining an upright posture with the waist and shoulders straight and feet spread shoulder-width apart, facing forward. The subject began with the dominant hand, and the arm was allowed to hang naturally without bending the elbow or wrist; the test was subsequently performed with the non-dominant hand. The measurement was performed three times for each hand with a one-minute interval between repetitions, allowing for rest. We analyzed the associations between various HGS indices and depression. First, we utilized HGS with the dominant hand (dominant-HGS) and with both hands (both-HGS) as markers of absolute HGS. Dominant-HGS represents the maximum HGS value among three measurements taken on the dominant hand, while both-HGS is the average HGS value from three measurements on each hand. Additionally, relative HGS indices were calculated as dominant-HGS and both-HGS divided by height, weight, BMI, WC, and the WHtR. Anthropometric measurements, including height, weight, and WC, were taken by trained medical personnel following standardized protocols. Height was measured using a Seca 225 portable stadiometer (Seca, Hamburg, Germany) with a precision of 0.1 cm. Weight was measured using an electronic scale (GL-6000-20; G-Tech International, Seoul, Korea) with a precision of 0.1 kg. WC was measured using a SECA 200 (Seca, Hamburg, Germany) tape measure with a precision of 0.1 cm, and measurements were taken at the midline between the lower rib margin and iliac chest. BMI was calculated as weight (kg) divided by the square of height (m^2^). The WHtR was calculated as waist circumference divided by height. Blood pressure was measured three times by a standard mercury sphygmomanometer (Baumanometer Wall Unit 33(0850), USA). The measurements were taken after the participants had rested for 5 min in a seated position with the heart and arm at the same height. Subsequently, the average of the second and third measurements was calculated.

### 2.4 Covariates

The selection of relevant covariates was based on literatures. We utilized sociodemographic variables such as education level, occupation, household income, health-related behavioral factors, and chronic disease-related variables as covariates. Age was used as a covariate (7, 8, 14, 15, 17, 18). Education level was classified into four categories (7, 9, 10, 15, 17, 18), while occupation was divided into seven categories (9, 17, 18). Household income was classified into four categories (quartiles) based on the average monthly household income (7, 14, 15, 17, 18). Stress was divided into four groups (extremely, very, slightly, and rarely) due to the degree of perceived stress (8, 13). Alcohol consumption was classified into two categories based on drinking status in the past year (9, 14, 15, 17, 18), and smoking status was classified into four categories based on the degree of smoking (7–10, 14, 17, 18). Resistance exercise was classified into four categories based on the response to the question (7–10, 14, 15, 17, 18) “How many days did you engage in strength exercises such as push-ups, sit-ups, weightlifting, gymnastics, or pull-ups in the past week?” Menopause was classified into two categories based on whether menopause had occurred (10, 17, 18). Chronic disease variables, such as hypertension, diabetes status, hypercholesterolemia status, and hypertriglyceridemia status, were based on the results of health screening examinations and health survey responses (7–10, 14, 15). Hypertension was defined as self-reported current use of antihypertensive medication, systolic blood pressure (SBP) ≥ 140 mmHg, or diastolic blood pressure (DBP) ≥ 90 mmHg. Diabetes status was based on doctor-diagnosed diabetes status, self-reported current use of antidiabetic medication, or fasting plasma glucose ≥126 mg/dL. Hypercholesterolemia was defined as a total cholesterol level ≥ 240 mg/dL or self-reported current use of cholesterol-lowering medications. Hypertriglyceridemia was based on a triglyceride level ≥ 200 mg/dL.

### 2.5 Statistical analysis

All analyses in this study were conducted using SPSS 28 (IBM SPSS, Inc., Chicago, IL, USA) for Windows following the guidelines for utilizing raw data from the KDCA’s National Health and Nutrition Examination Survey. Subjects were classified into male and female groups and then further classified into non-depression and depression groups; the characteristics of these groups were described with percentages and standard errors for categorical variables and means and standard errors for continuous variables. For the analysis of sex differences, t tests based on a general linear model were used for continuous variables, and Rao–Scott chi-square tests were employed for categorical variables. The characteristics of the study subjects can be found in Table 1. The associations between depression and anthropometric indices and between depression and HGS were analyzed through complex sample binary logistic regression models after the data were standardized. The multicollinearity between variables in the binary logistic regression model was assessed using the variance inflation factor (VIF), and the linearity between the logit of the dependent variable and the independent variables was verified through the Box-Tidwell test. Three models were created based on adjustment variables: the crude model included no adjustments; Model 1 included adjustments for age; and Model 2 included adjustments for age, education level, occupation, household income, stress, alcohol consumption, smoking status, resistance exercise, chronic disease, and menopause (only in women).

**Table 1:** Demographic characteristics of the subjects in this study.

Variables	Men		*p*-value	Women		*p* value
	Non-depression	Depression		Non-depression	Depression	
Subjects (*n*)	8,754	205		10,949	741	
Age (years)^***^	51.14 ± 0.21	54.71 ± 1.15	0.002	51.83 ± 0.19	56.11 ± 0.63	<0.001
Residential area^**^			0.843			0.011
Urban	83.22 (1.15)	83.78 (2.86)		84.81 (1.05)	80.89 (1.97)	
Rural	16.78 (1.15)	16.22 (2.86)		15.19 (1.05)	19.11 (1.97)	
Education level^***^			<0.001			<0.001
<= Elementary school	11.50 (0.40)	19.57 (3.10)		21.25 (0.55)	32.85 (1.94)	
Middle school	9.70 (0.40)	15.20 (3.04)		9.77 (0.35)	14.54 (1.53)	
High school	30.40 (0.60)	36.12 (3.90)		31.84 (0.59)	31.25 (2.01)	
> = University	48.30 (0.80)	29.11 (4.03)		37.14 (0.72)	21.36 (1.85)	
Occupation^***^			<0.001			<0.001
White-collar worker	19.90 (0.60)	12.60 (2.83)		12.94 (0.41)	7.12 (1.23)	
Office worker	15.20 (0.50)	6.04 (2.22)		9.31 (0.34)	5.71 (1.06)	
Service worker	10.50 (0.40)	7.87 (2.41)		15.20 (0.44)	9.89 (1.42)	
Farmer or fisher	4.90 (0.40)	6.50 (1.78)		2.56 (0.25)	2.41 (0.67)	
Blue-collar worker	22.80 (0.60)	17.21 (3.67)		2.81 (0.19)	2.59 (0.73)	
Basic occupations	6.80 (0.30)	7.20 (2.07)		9.11 (0.32)	10.88 (1.26)	
Unemployed (housewife, etc.)	20.00 (0.50)	42.58 (4.03)		48.07 (0.61)	61.40 (2.13)	
Household income^***^			<0.001			<0.001
Low	12.50 (0.40)	29.34 (3.69)		17.16 (0.51)	30.79 (1.99)	
Middle-low	24.00 (0.60)	28.78 (3.84)		24.24 (0.54)	27.21 (1.91)	
Middle-high	30.70 (0.60)	23.21 (3.73)		28.96 (0.57)	20.17 (1.78)	
High	32.90 (0.80)	18.67 (3.58)		29.64 (0.72)	21.84 (1.80)	
Alcohol consumption status^***^			<0.001			<0.001
No	15.70 (0.50)	31.71 (3.79)		65.80 (0.60)	57.19 (2.05)	
Yes	84.30 (0.50)	68.29 (3.79)		34.20 (0.60)	42.81 (2.05)	
Smoking status^***^			0.104			<0.001
Daily	35.40 (0.70)	43.00 (4.27)		4.58 (0.26)	8.30 (1.30)	
Former	42.80 (0.70)	34.61 (3.88)		5.68 (0.26)	7.60 (1.10)	
Never	21.80 (0.50)	22.38 (3.37)		89.74 (0.36)	84.10 (1.60)	
Stress level^**^			<0.001			<0.001
Extremely stressed	3.30 (0.20)	12.99 (2.81)		3.90 (0.20)	13.84 (1.60)	
Very stressed	21.20 (0.50)	35.68 (4.02)		21.20 (0.50)	32.37 (1.97)	
Slightly stressed	58.90 (0.60)	41.41 (3.98)		58.60 (0.60)	43.81 (2.15)	
Rarely stressed	16.70 (0.40)	9.93 (2.51)		16.20 (0.40)	9.97 (1.19)	
Resistance exercise frequency^***^			0.160			0.186
Never	67.40 (0.60)	73.29 (3.85)		83.30 (0.44)	85.39 (1.48)	
1 ~ 2 day per week	10.70 (0.40)	5.07 (1.91)		6.69 (0.28)	5.58 (0.98)	
3 ~ 4 day per week	10.70 (0.40)	9.39 (2.56)		5.84 (0.28)	4.12 (0.75)	
> = 5 day per week	11.20 (0.40)	12.26 (2.56)		4.17 (0.21)	4.91 (0.92)	
Menopausal status			–			<0.001
No	–	–		49.94 (0.69)	35.45 (2.16)	
Yes	–	–		50.06 (0.69)	64.55 (2.16)	
Hypertension status^***^			0.073			0.001
No	65.62 (0.62)	57.70 (4.23)		73.10 (0.50)	65.81 (1.96)	
Yes	34.38 (0.62)	42.30 (4.23)		27.00 (0.50)	34.19 (1.96)	
Diabetes status ^***^			0.139			<0.001
No	86.10 (0.40)	80.72 (2.89)		90.08 (0.32)	84.02 (1.46)	
Yes	13.80 (0.40)	19.28 (2.89)		9.92 (0.32)	15.98 (1.46)	
Hypercholesterolemia status ^***^			0.018			<0.001
No	80.22 (0.48)	72.39 (3.68)		76.66 (0.47)	65.40 (2.00)	
Yes	19.78 (0.48)	27.61 (3.68)		23.34 (0.47)	34.60 (2.00)	
Hypertriglyceridemia status ^***^			0.001			0.008
No	76.68 (0.52)	65.31 (3.92)		90.37 (0.34)	88.15 (1.32)	
Yes	23.32 (0.52)	34.69 (3.92)		9.63 (0.34)	11.85 (1.32)	
Blood pressure						
SBP (mmHg)^***^	120.54 ± 0.20	122.26 ± 1.17	0.141	116.54 ± 0.22	118.00 ± 0.71	0.048
DBP (mmHg)^***^	78.66 ± 0.14	79.58 ± 0.85	0.282	74.03 ± 0.12	74.00 ± 0.39	0.939
Anthropometrics						
Height (cm)^***^	170.81 ± 0.09	169.19 ± 0.50	0.001	157.61 ± 0.08	156.15 ± 0.28	<0.001
Weight (kg)^***^	71.96 ± 0.15	72.05 ± 0.94	0.917	58.31 ± 0.11	58.42 ± 0.42	0.807
BMI (kg/m^2^)^***^	24.60 ± 0.04	25.11 ± 0.28	0.069	23.49 ± 0.04	23.94 ± 0.15	0.003
WC (cm)^***^	87.02 ± 0.11	88.88 ± 0.65	0.005	79.47 ± 0.13	80.87 ± 0.42	0.001
WHtR ^***^	0.51 ± 0.00	0.53 ± 0.00	<0.001	0.51 ± 0.00	0.52 ± 0.00	<0.001
Absolute hand grip strength						
Dominant-HGS (kg)^***^	38.73 ± 0.11	35.48 ± 0.75	<0.001	22.77 ± 0.07	21.37 ± 0.22	<0.001
Both-HGS (kg)^***^	37.7 ± 0.11	34.22 ± 0.74	<0.001	22.05 ± 0.07	20.67 ± 0.22	<0.001
Relative hand grip strength						
Dominant-HGS/BMI (kg/BMI)^***^	1.59 ± 0.00	1.43 ± 0.03	<0.001	0.99 ± 0.00	0.91 ± 0.01	<0.001
Dominant-HGS/WC (kg/WC)^***^	0.45 ± 0.00	0.40 ± 0.01	<0.001	0.29 ± 0.00	0.27 ± 0.00	<0.001
Dominant-HGS/WHtR (kg/WHtR)^***^	76.79 ± 0.25	68.23 ± 1.53	<0.001	46.03 ± 0.18	42.09 ± 0.52	<0.001
Both-HGS/BMI (kg/BMI)^***^	1.55 ± 0.00	1.38 ± 0.03	<0.001	0.96 ± 0.00	0.88 ± 0.01	<0.001
Both-HGS/WC (kg/WC)^***^	0.44 ± 0.00	0.39 ± 0.01	<0.001	0.28 ± 0.00	0.26 ± 0.00	<0.001
Both-HGS/WHtR (kg/WHtR)^***^	74.78 ± 0.24	65.81 ± 1.49	<0.001	44.6 ± 0.18	40.73 ± 0.51	<0.001

SBP, systolic blood pressure; DBP, diastolic blood pressure; HGS, handgrip strength; BMI, body mass index; WC, waist circumference; WHtR, waist-to-height ratio. Dominant-HGS refers to the maximum handgrip strength measured in the dominant hand, while Both-HGS represents the average handgrip strength measured in both hands. Continuous data are presented as the means ± SEs (standard errors), and categorical data are presented as percentages (SEs). *p < 0.05, **p < 0.01, ***p < 0.001. *, **, and *** Represent *p* values for sex differences between all men and women. *p* values were obtained from Rao–Scott chi-square tests for categorical variables and from a general linear model for continuous variables between the depression group and the non-depression group.

## 3 Results

### 3.1 Patient demographic characteristics

Table 1 presents the demographic characteristics of all the variables used in this analysis. The final study subjects consisted of 20,649 adults aged 30 years and older, comprising 8,959 men (43.4%) and 11,690 women (56.6%). The prevalence of depression among adults aged 30 years and older in South Korea was 4.58%, with rates of 2.29% for men and 6.34% for women. This finding indicates that the prevalence of depression among women is 2.76 times greater than that among men. Among the sociodemographic variables, such as age, residential area, education level, occupation, and household income, excluding residential area for men (*p* = 0.843), all the variables exhibited significant differences between the non-depression and depression groups for both men and women. For both sexes, compared with individuals in the non-depression group, those in the depression group tended to be older, have a lower education level, have a higher unemployment rate, and have lower household income. Residential area significantly differed only for women, with the depression group indicating a greater proportion of rural residents than the non-depression group. Specifically, within the four household income categories, the ‘low’ category had a greater prevalence, being 2.34 times greater among men and 1.79 times greater among women in the depression group than among those in the non-depression group. Among the health-related variables, such as alcohol consumption, smoking status, stress, and resistance exercise, alcohol consumption and stress were significantly different between the two groups of men. Among women, significant differences were found in alcohol consumption, smoking status, and stress, but not resistance exercise (*p* = 0.186), between the two groups. Compared to those in the non-depression group, men in the depression group exhibited less alcohol consumption, while women showed greater alcohol consumption. Smoking status significantly differed only for women, with the ‘Daily’ smoking rate being 1.81 times greater in the depression group than in the non-depression group. Notably, the ‘extremely’ stressed category was significantly more common in the depression group than in the non-depression group, with a 3.9-and 3.54 fold increase in depression prevalence among men and women in that stress category, respectively. Additionally, the rate of menopause was greater in the depression group than in the non-depression group. Among chronic health conditions such as hypertension, diabetes, hypercholesterolemia, and hypertriglyceridemia, men exhibited significant differences in the prevalence of hypercholesterolemia and hypertriglyceridemia between the two groups, with the prevalence being greater in the depression group than in the non-depression group. Among women, all chronic conditions exhibited significant differences between the two groups; notably, the prevalence of diabetes in the depression group was 1.61 times greater than that in the non-depression group. Blood pressure-related variables did not significantly differ between the two groups of men; however, for women, only SBP significantly differed between the depression and non-depression groups. Among the anthropometric measurements, there were significant differences in height, WC, and the WHtR between the two groups of men, but not for weight (*p* = 0.917) and BMI (*p* = 0.069). For women, all anthropometric variables, except for weight (*p* = 0.807), exhibited significant differences between the two groups. All HGS indices exhibited significant differences between the two groups for both men and women. In both sexes, HGS in the depression group tended to be lower than that in the non-depression group.

### 3.2 Associations of depression with anthropometric indices and HGS

Tables 2, 3 illustrate the associations of depression with HGS and anthropometric indices in Korean adults aged 30 years and older. In men, both-HGS/BMI (odds ratio (OR) = 0.58 [0.49–0.69], *p* < 0.001) and both-HGS/WHtR (OR = 0.58 [0.48–0.69], *p* < 0.001) exhibited slightly greater negative associations with depression than did the other indices according to the crude analysis. According to the models adjusted for covariates, both-HGS/WHtR (OR = 0.68 [0.55–0.83], adjusted *p* < 0.001 in Model 2) exhibited a slightly stronger negative association with depression than did both-HGS/WC (OR = 0.69 [0.57–0.82], adjusted *p* < 0.001 in Model 2). In women, all HGS indices showed similar associations with depression in all crude analyses and in Model 1 and Model 2. However, anthropometric indices such as height, weight, BMI, WC, and the WHtR were less strongly associated with depression than were all the HGS indices in both sexes.

**Table 2:** Associations of depression with absolute and relative HGS indices in men.

Variables	Crude		Model 1		Model 2	
	OR (95% CI)	*p-*value	Adj. OR (95% CI)	Adj. *p-*value	Adj. OR (95% CI)	Adj. *p-*value
Age	1.31 (1.11–1.55)	0.002				
Anthropometrics						
Height	0.78 (0.67–0.91)	0.001	0.86 (0.72–1.03)	0.093	0.96 (0.81–1.14)	0.640
Weight	1.01 (0.86–1.18)	0.917	1.13 (0.96–1.33)	0.132	1.14 (0.97–1.34)	0.107
BMI	1.16 (1.00–1.35)	0.056	1.22 (1.05–1.42)	0.011	1.18 (1.01–1.38)	0.040
WC	1.23 (1.07–1.41)	0.004	1.23 (1.07–1.42)	0.003	1.17 (1.01–1.37)	0.040
WHtR	1.34 (1.17–1.54)	<0.001	1.30 (1.12–1.51)	0.001	1.19 (1.02–1.40)	0.032
Absolute hand grip strength						
Dominant-HGS	0.65 (0.54–0.79)	<0.001	0.67 (0.54–0.84)	0.001	0.77 (0.62–0.95)	0.014
Both-HGS	0.63 (0.52–0.76)	<0.001	0.64 (0.51–0.80)	<0.001	0.74 (0.60–0.91)	0.004
Relative hand grip strength						
Dominant-HGS/BMI	0.60 (0.51–0.72)	<0.001	0.62 (0.52–0.75)	<0.001	0.71 (0.59–0.85)	<0.001
Dominant-HGS/WC	0.60 (0.50–0.72)	<0.001	0.62 (0.50–0.76)	<0.001	0.71 (0.58–0.87)	0.001
Dominant-HGS/WHtR	0.60 (0.50–0.71)	<0.001	0.60 (0.48–0.75)	<0.001	0.70 (0.57–0.87)	0.001
Both-HGS/BMI	0.58 (0.49–0.69)	<0.001	0.60 (0.50–0.72)	<0.001	0.69 (0.57–0.82)	<0.001
Both-HGS/WC	0.59 (0.49–0.70)	<0.001	0.59 (0.48–0.73)	<0.001	0.69 (0.56–0.84)	<0.001
Both-HGS/WHtR	0.58 (0.48–0.69)	<0.001	0.57 (0.46–0.71)	<0.001	0.68 (0.55–0.83)	<0.001

HGS, handgrip strength; BMI, body mass index; WC, waist circumference; WHtR, waist-to-height ratio; OR, odds ratio; CI, confidence interval. OR and *p* values were derived from the crude and adjusted models using complex sample binary logistic regression. Odds ratios were estimated with 95% confidence intervals. Dominant-HGS refers to the maximum handgrip strength measured in the dominant hand, while Both-HGS represents the average handgrip strength measured in both hands. Model 1 was adjusted for age. Model 2 was adjusted for education level, occupation, household income, stress, alcohol consumption, smoking status, resistance exercise, chronic disease, and age.

**Table 3:** Associations of depression with absolute and relative HGS indices in women.

Variables	Crude		Model 1		Model 2	
	OR (95% CI)	*p-*value	Adj. OR (95% CI)	Adj. *p-*value	Adj. OR (95% CI)	Adj. *p-*value
Age	1.36 (1.24–1.48)	<0.001				
Anthropometrics						
Height	0.80 (0.73–0.87)	<0.001	0.92 (0.82–1.02)	0.124	0.94 (0.84–1.05)	0.299
Weight	1.01 (0.92–1.11)	0.807	1.04 (0.95–1.14)	0.383	1.00 (0.91–1.10)	0.963
BMI	1.13 (1.05–1.22)	0.002	1.07 (0.98–1.17)	0.107	1.02 (0.93–1.12)	0.710
WC	1.15 (1.06–1.25)	0.001	1.05 (0.95–1.15)	0.331	0.97 (0.88–1.07)	0.515
WHtR	1.22 (1.12–1.32)	<0.001	1.07 (0.97–1.18)	0.164	0.98 (0.88–1.09)	0.714
Absolute hand grip strength						
Dominant-HGS	0.76 (0.69–0.82)	<0.001	0.83 (0.76–0.92)	<0.001	0.87 (0.79–0.96)	0.006
Both-HGS	0.76 (0.69–0.82)	<0.001	0.84 (0.76–0.92)	<0.001	0.88 (0.80–0.97)	0.011
Relative hand grip strength						
Dominant-HGS/BMI	0.73 (0.67–0.79)	<0.001	0.80 (0.73–0.89)	<0.001	0.86 (0.78–0.96)	0.005
Dominant-HGS/WC	0.73 (0.67–0.79)	<0.001	0.81 (0.73–0.90)	<0.001	0.88 (0.79–0.98)	0.019
Dominant-HGS/WHtR	0.72 (0.66–0.79)	<0.001	0.80 (0.72–0.89)	<0.001	0.87 (0.78–0.97)	0.016
Both-HGS/BMI	0.73 (0.67–0.79)	<0.001	0.80 (0.73–0.89)	<0.001	0.87 (0.79–0.96)	0.008
Both-HGS/WC	0.73 (0.67–0.80)	<0.001	0.81 (0.73–0.90)	<0.001	0.89 (0.80–0.99)	0.033
Both-HGS/WHtR	0.72 (0.66–0.79)	<0.001	0.80 (0.72–0.89)	<0.001	0.88 (0.79–0.99)	0.027

BMI, body mass index; WC, waist circumference; WHtR, waist-to-height ratio; HGS, handgrip strength; OR, odds ratio; CI, confidence interval. OR and *p-*values were derived from the crude and adjusted models using complex sample binary logistic regression. Odds ratios were estimated with 95% confidence intervals. Dominant-HGS refers to the maximum handgrip strength measured in the dominant hand, while Both-HGS represents the average handgrip strength measured in both hands. Model 1 was adjusted for age. Model 2 was adjusted for education level, occupation, household income, stress, alcohol consumption, smoking status, resistance exercise, chronic disease, menopausal status, and age.

## 4 Discussion

In this study, we demonstrated that low absolute and relative HGS were negatively associated with depression in both men and women. Additionally, in men, relative HGS indices from the dominant hand and both hands had stronger associations with depression than did absolute HGS indices, but in women, the associations were similar for both absolute and relative HGS indices. The magnitude of the association between HGS and depression was greater in men than in women. All the anthropometric indices showed a weaker association with depression than did the absolute and relative HGS indices.

Many studies have been conducted to reveal the association between depression and HGS in many countries (6–15). Marques et al. (6) examined the association between HGS and depression symptoms in European adults. They reported that higher HGS was associated with a decreased risk of depression and argued that there was an interrelationship between depression and physical strength. Ashdown-Franks et al. (7) investigated the relationship between HGS and depression based on 34,129 subjects from six low-and middle-income countries. They found that lower HGS was related to greater odds of depression, and the association between HGS and depression did not differ by sex. Fukumori et al. (8) tested the association between HGS and the risk of depression in a longitudinal prospective cohort study in Japan and reported that subjects with lower HGS had a greater risk of depression and that low HGS was related to the longitudinal development of depression symptoms after 1 year. Gu et al. (9) assessed the relationship between HGS and depressive symptoms in a large population in China and reported that HGS was negatively associated with depressive symptoms and that the association was greater in women than in men. Smith et al. (10) examined the relationship between HGS and depressive symptoms according to weight status in U.S. adults. They argued that women with a moderate or high number of depressive symptoms had a 1.60 kg lower HGS than did those with few or no symptoms. Additionally, Firth et al. (11) examined the association of HGS with various brain region volumes and white matter hyperintensities between a depressive disorder group and a healthy control group in the UK. They reported that higher HGS was related to increased hippocampal volume, and the association between HGS and hippocampal volume was greater in the depressive disorder group than in the healthy control group. Lian et al. (12) tested the bidirectional relationship between HGS and depressive symptoms in China and reported that low HGS was associated with a subsequently high risk of depressive symptoms. The authors emphasized the need for simultaneous physical and mental health interventions since they found a linear dose–response association between HGS and the risk of depressive symptoms. In Korea, Han et al. (13) investigated the association between HGS and depression according to different socioeconomic levels in older adults. They reported that older adults with a low income level had a greater association between HGS and depressive symptoms than did those with a high income level. Additionally, Kang et al. (14) examined the relationship between HGS and high-sensitivity C-reactive protein levels in young, middle-aged, and older adults with depressive disorders. They documented that subjects with depressive disorder tended to have decreased HGS in all age groups, and the association between low HGS and high levels of high-sensitivity C-reactive protein was significant in older adults. Our findings are consistent with the results of previous studies, which indicated that low HGS was negatively associated with depression (6–10, 12, 14, 15), and the association between HGS and depression was greater in men than in women (9), unlike the results of previous studies (7). Additionally, our findings are consistent with the results of previous studies, indicating that low HGS was more negatively related to depression in subjects with low socioeconomic or income levels (13).

However, the underlying mechanism of the association between HGS and depression is unclear. The cause of depression is complex due to the combined effects of various factors, including sociodemographic and socioeconomic factors, such as income level, education level, and social isolation; biological factors, such as inflammation; psychological factors, such as the effects of bereavement or illness; and family environmental factors, such as distressing family events (13, 15, 23). However, one potential mechanism or explanation may be that low HGS and biological aging induce high functional limitations and physical disability (10, 15, 24–26), and the combination of high limitations and disability with low quality of life may lead to depressive symptoms and cognitive impairment (7, 10, 15, 25, 27). For example, high physical activity was associated with a reduced risk of depression and antidepressant effects in adults (7, 15, 28, 29). On the other hand, inflammatory factors are common risk factors for both low HGS and depression (15). High levels of inflammatory cytokines such as C-reactive protein may induce low muscle strength (15), and there is a significant association between low HGS and low-grade inflammation, which is indicated by the independent association between elevated levels of high-sensitivity C-reactive protein and low HGS in older people with depression (14, 23). Additionally, muscle-derived cytokines (myokines) are linked to brain function (9), having been shown to improve cognition and memory (30). Finally, cytokines may lead to depressive symptoms through changes in neurotransmitters (15), and inversely, depression may lead to increased inflammation (23). We assume that these associations may be risk factors and markers of depression rather than causes of depression. Therefore, further studies are needed to found the complex mechanism underlying the association between muscle strength and depression, which is likely influenced by interactions among many biological factors, social characteristics, and psychological factors.

This study has several limitations. We cannot establish a causal relationship between HGS and depression due to the cross-sectional design of this study. The data used in this study were collected via a health interview survey administered via questionnaires. For the diagnosis of depression, the 9-item Patient Health Questionnaire (PHQ-9) (31) was included in the KNHANES data. However, PHQ-9 information was only included in 2014, 2016, and 2018. Therefore, we used physicians’ diagnosis of depression through interviews to analyze data from 2014 to 2019. There may be respondent recall bias in collecting diagnosis information through interviews. To avoid respondent recall bias, the survey involved a face-to-face interview accompanied by well-trained staff or experts according to rigorous guidelines, and several previous studies used the same classification method for depression as we did (32, 33). In this study, we used only two absolute HGS indices such as the maximum HGS value among three measurements taken on the dominant hand and the average HGS value from three measurements on each hand. In studies utilizing HGS, various absolute HGS indices are created and analyzed. Actually, the definition of absolute HGS index is used differently across studies in terms of measured hand (dominant hand, non-dominant hand, and both hands), the number of measurements, and the maximum or average values among the measured values. Therefore, future research is needed to determine which HGS index is superior for a specific disease. Finally, the results in this study have statistical limitations because the present study did not consider the statistical significance according to a large sample size. Even though this study had these limitations, the statistical results were robust due to the use of the very large KNHANES dataset, which is a nationally representative sample of the Korean adult population.

In conclusion, depressive disorder is one of the leading mental health problems worldwide. Absolute and relative HGS may be potential indicators of depression, and a weaker relative HGS may be more likely to associate with mental health status than absolute HGS in men. To our knowledge, this is the first report on the association of depression with absolute and relative HGS indices (i.e., HGS combined with WC and the WHtR) in a large population.

## Data Availability

Publicly available datasets were analyzed in this study. This data can be found here: the data used in the present study are available from the Korea National Health and Nutrition Examination Survey (KNHANES) collected by the Korea Centers for Disease Control and Prevention (KCDC). Anyone can freely access the data (https://knhanes.kdca.go.kr/knhanes/sub03/sub03_02_05.do).
